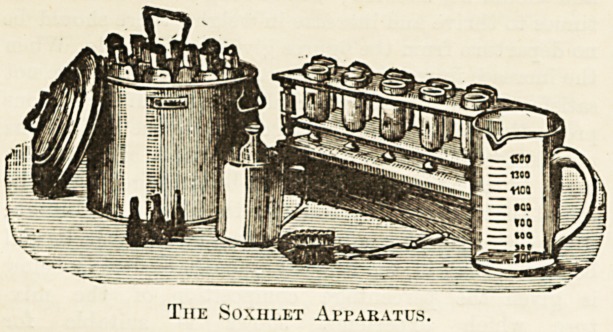# The Artificial Feeding of Infants.—A Rational Method

**Published:** 1900-03-24

**Authors:** Eric Pritchard


### HOSPITAL CLINICS AND MEDICAL PROGRESS.

### THE ARTIFICIAL FEEDING OF INFANTS.— A RATIONAL METHOD.

By ERIC PRITCHARD, M.A., M.D. (Oxon).

IN America, owing chiefly to the energy of Holt and Rotch, the subject of artificial feeding for infants has during the last few years received the attention which its importance undoubtedly deserves, and for the old haphazard methods of inaccuracy and carelessness they have substituted one of scientific exactness. They have demonstrated that the want of success which has heretofore attended the methods in vogue has been as much due to the absolute disregard of the infants' physiological requirements as to mistakes in the preparation and supply of the food itself. They condemn the use of all proprietary foods, and insist on the substitution of properly-modified cow's milk in all cases in which the infant cannot be breast-reared. They show that by appropriate treatment cow's milk can be so modified as to approximate very closely to the human standard; and indeed to any other required standard, when, owing to natural idiosyncrasies or other reasons, a departure from the normal is indicated. To modify cow's milk to any particular standard it is only necessary to pay attention to the relative proportions of fat, proteid, and sugar; by simple means a milk mixture can be prepared containing these three elements in any required proportion, and a table has been constructed which contains the percentages which are applicable for infants of all ages, and tally with those of human milk at the corresponding stage of lactation. In ordering food for an infant of any particular age the table must be referred to, the percentage composition written down in the form of a prescription and sent to a dairy which understands the making up of such prescriptions. In America there are special dairies for this purpose. They are known as the Walker-Gordon milk laboratories. They have been established in most of the large centres of population, and in London there has also been one for a considerable number of years. Here in England the system has not been generally adopted, partly because the method is an expensive one, and partly because we are not familiar with the figures and percentages which must necessarily be known if we are to write out the prescription for a modified milk. The object of this paper is to show that it is not only possible but comparatively easy to dispense with the services of a regular milk laboratory, and thus eliminate the objection on the ground of expense, and further to supply a series of tables and directions for the home preparation of a milk mixture according to prescription, and thus familiarise those interested in the subject with the necessary essentials for understanding the method.

Now in the ordinary method of feeding by prescription and where a milk laboratory is at hand to supply the mixture, the proportions are made out in "percentages" according to a fixed table of quantities corresponding to the age of the infant. For instance, the prescription for an infant of three weeks in normal health and of average weight will run as follows:—

| | | |
|---|---|---|
| ℞.—Fat | 3·00 | per cent. |
| Sugar | 6·00 | " " |
| Proteids | 1.00 | " " |

Reaction slightly alkaline.

To be heated to 167 deg. Fahr.

To be made up into 10 bottles, each containing 1 ounce and 6 drachms, making a total of 18 ounces for the 24 hours. It is easy enough for a milk laboratory, with the necessary knowledge and appliances to make up a mixture of such percentage composition. But to anybody who has not studied the subject it would present insuperable difficulties to do so at home. In fact, to convert a prescription given in percentages into everyday weights and measures is an arithmetical problem of no mean order. The combination of milk, cream, milk sugar, and water, which results in a mixture in which the percentages of fat, sugar, and proteid are as given in the above prescription, can only be worked out by a person familiar with the handling of such problems. It is possible, however, to supply a table for the conversion of such prescriptions, and by the use of such a table the process is perfectly simple and easy in the hands of any competent person. Such a table I have constructed, and supply a little further on. Roughly speaking I have taken the mean of Holt's and Rotch's prescription as the standard for my quantities, but have made such modification as I have found from practical experience to be of advantage. The table is intended only as a proximate guide under normal conditions of health, weight, &c.; under other circumstances modifications may be, and should be, effected; but so long as the infant continues to thrive and increase in weight there should be no departure from the figures given in the table. When the increase in weight and the general progress are not satisfactory modifications should be made in the various proportions of the mixture according to the indications until success shows that further changes are unnecessary. If the accompanying table be examined it will be found that the top line contains the age of the infant; if the corresponding column be read downwards it will be found that in the vertical space immediately below is given the percentage composition of the mixture which experience shows is suitable for such an age. In the spaces below this will be found in order the various quantities of milk, cream, sugar, lime water, and water, which are necessary to add to make up such a percentage composition. For instance, if we wish to make up a suitable mixture for a child one week old, we take the third column of the table. In the first place, we notice that such a mixture should be of the following percentage composition, namely: fat, 2·5 per cent.; proteid, 1·0 per cent.; sugar, 6 per cent. Now to make such a percentage mixture sufficient for 24 hours we must take one ounce and four drachms of milk, one ounce and four drachms of cream, one ounce and two drachms of sugar, half-an-ounce of lime water. Mix them up together, and add water till the total quantity reaches 12 ounces. This

Table for Preparing Modified Milk for a Healthy Infant of Average Weight During the Successive Stages of Its Life From One Day to One Year.

(The milk should be ordinary mixed milk and the cream contain 16 per cent. fat. Ordinary good cream obtained by skimming may be assumed to be of this strength.)

| Age of Infant. | | 1st day. | 2nd day. | 3rd-14th day. | 14th-28th day. | 2nd month. | 3rd month. | 4th-6th month. | 6th-8th month. | 8th-12th month. |
|---|---|---|---|---|---|---|---|---|---|---|
| Percentage Composition of mixture. | Fat ... ... | 0·0 | 2·0 | 2·5 | 3·0 | 3·5 | 4·0 | 4·0 | 4·0 | 4·0 |
| | Proteid ... | 0·0 | 0·5 | 1·0 | 1·0 | 1·5 | 1·5 | 1·5 | 2·0 | 2 5 |
| | Sugar ... | 5·0 | 5·0 | 6·0 | 6·0 | 6·0 | 6·5 | 7·0 | 7·0 | 7·0 |
| | Lime water.. | 0·0 | 5·0 | 5·0 | 5·0 | 5·0 | 5·0 | 5·0 | 5·0 | 5·0 |
| Milk ... ... ... ... | | None | None | 1 oz. 4 dr. | 1 oz. 4 dr. | 4 oz. 6 dr. | 4 oz. 2 dr. | 5 oz. 4 dr. | 13 oz. 4 dr. | 23 oz. |
| Cream (16 per cent. fat) ... | | None | 1 oz. | 1 oz. 4 dr. | 3 oz. | 4 oz. 2 dr. | 6 oz. 6 dr. | 8 oz. | 7 oz. 4 dr. | 7 oz. |
| Milk sugar (by measure, *not* by weight)... ... ... | | 4 dr. | 6 dr. | 1 oz. 2 dr. | 1 oz. 6 dr. | 2 oz. | 3 oz. | 3 oz. 4 dr. | 4 oz. | 4 oz. |
| Lime water ... ... ... | | None | 3 dr. | 4 dr. | 6 dr. | 1 oz. | 1 oz. 4 dr. | 2 oz. | 2 oz. 4 dr. | 3 oz. |
| Total quantity for 24 hours (made up by add. of water) | | 5 oz. | 8 oz. | 12 oz. | 18 oz. | 24 oz. | 30 oz. | 36 oz. | 42 oz. | 48 oz. |
| Quantity in each bottle (for one feeding) ... ... | | 4 dr. | 6½ dr. | 1 oz. 1 dr. | 1 oz. 6 dr. | 3 oz. | 3 oz. 5 dr. | 4 oz. 4 dr. | 6 oz. | 8 oz. |
| Total number of bottles ... | | 10 | 10 | 10 | 10 | 8 | 8 | 8 | 7 | 6 |
| Intervals between feedings.. | | 2¼ hours | 2¼ hours | 2¼ hours | 2¼ hours | 3 hours | 3 hours | 3 hours | 3½ hours | 4 hours |

mixture must be divided into 10 equal parts, and poured into 10 bottles. Each bottle will contain one ounce and one drachm of the mixture, sufficient for one feed. Or again, in the next column, for a child from 14 days to one month old. The percentage composition should be: Fat, 3 per cent.; proteid, 1 per cent.; sugar, 6 per cent.; and to make up such a mixture we must take of milk, 1 ounce 4 drachms; of cream, 3 ounces; of sugar, 1 ounce 6 drachms; of lime water, 6 drachms; and add water till the total measures 18 ounces; this must be divided into 10 bottles of 1 ounce 6 drachms. After having prepared the appropriate milk mixture, the next stage is to sterilise the food, and for this purpose the most convenient apparatus is that known as the Soxhlet, of which an illustration is appended. The complete apparatus consists

The Soxhlet Apparatus.

of: (1) A metal saucepan with lid, fitted with (2) strainer, to hold 10 bottles; (3) a stand for 10 bottles, with drawer; for keeping extra parts of the apparatus; (4) 20 feeding bottles; (5) 12 rubber discs for sealing up bottles; (6) 10 metal caps for holding discs in position; (7) three rubber nipples; (8) glass measures; (9) brush for cleaning bottles; (10) glass funnel. The price of the apparatus is £1 1s., and, in ordering the same, it must be specially stated that glass measures graduated in ounces and drachms are required, otherwise measures graduated in litres and gram will be supplied, as the apparatus is made on the Continent and chiefly used there. It must also be stated that a glass funnel is required, as this item is not usually included in the list. The complete apparatus with measures and funnel may be obtained from Alfred Cox, 108, New Bond Street, W. The method of using the apparatus is as follows: Having prepared the appropriate milk mixture, it has to be divided equally among the bottles that will be required for the 24 hours (the number is stated in the last space but two in the corresponding column). The amount required for each bottle is stated in the last space but one. This quantity must be measured out in the small glass measure and poured into one of the bottles. The other bottles should then be placed in a row and filled up to the same level, the glass funnel being used for the filling. When the necessary bottles have been filled, each must be covered with an india-rubber disc, and a metal cap slipped over the neck of the bottle to hold the disc in place. All the bottles are then placed in the metal strainer, and put into the saucepan, which has previously been filled about one-third of the way up with cold water. The saucepan is then covered with the lid and set over a spirit lamp, and kept there until it has been boiling for 20 minutes. It should then be removed, the lid lifted off, and allowed to cool. Subsequently the bottles are removed, and kept for use. They will be found securely sealed by the rubber discs, which are depressed into the openings of the bottles by atmospheric pressure. When a bottle is required for use the rubber disc is removed, and replaced by a nipple which slips over the neck of the bottle. The bottle and its contents are appropriately warmed in a can of hot water before each feeding. Full directions are supplied with the apparatus.

## Figures and Tables

**Figure f1:**